# Using pupae as appetitive reinforcement to study visual and tactile associative learning in the Ponerine ant *Diacamma indicum*

**DOI:** 10.1038/s41598-023-42439-w

**Published:** 2023-09-20

**Authors:** Parth Chandak, Udipta Chakraborti, Sumana Annagiri

**Affiliations:** Behaviour and Ecology Lab, Department of Biological Sciences, Indian Institute of Science Education and Research, Mohanpur, Kolkata, 741246 India

**Keywords:** Ecology, Evolution, Zoology

## Abstract

Associative learning is of great importance to animals, as it enhances their ability to navigate, forage, evade predation and improve fitness. Even though associative learning abilities of Hymenopterans have been explored, many of these studies offered food as appetitive reinforcement. In the current study, we focus on tactile and visual cue learning in an ant *Diacamma indicum* using a Y-maze setup with pupa as a positive reinforcement. Using pupa as a reward resulted in a significantly higher proportion of ants completing the training in a shorter time as compared to using food as reinforcement. Ants spent significantly more time in the conditioned arm for both visual cues (white dots or black dots) and tactile cues (rough or smooth surfaces) presented on the floor when associated with pupa, thus showing that they were capable of associative learning. On encountering a conflict between visual and tactile cues during the test, ants chose to spend significantly more time on the arm with the tactile cues indicating that they had made a stronger association between pupa and the tactile cue as compared to the visual cue during training. Using pupa as an ecologically relevant reward, we show that these solitary foraging ants living in small colonies are capable of visual and tactile associative learning and are likely to learn tactile cues over visual cues in association with pupa.

## Introduction

Learning is important for organisms that live in complex and “predictably unpredictable” environments^1^. For such organisms, it is important to integrate information from various senses and associate them, through experience in order to use stimuli as predictors of biologically relevant events in the environment^2^. Such learned associations offer a significant fitness advantage by maximising the probability of survival^1,3^. Associative learning has been the most intensively studied form of learning. It is the process through which organisms acquire information about relationships between events or entities in their environment. Associative learning has been studied in several species of invertebrates such as crabs^4,5^, nematodes^6^, flies^7^, cockroaches^8,9^, crickets^10^. Social insects such as honeybees, bumblebees and ants have been key models in studies on memory and learning. Visual learning has been documented in many species of ants. Ants use landmark panoramas for navigation^11–13^ and optic flow created using a pattern of stripes helps them to measure distance^14,15^. They are also able to discriminate between different wavelengths of light and different intensities of light of the same wavelength^16^.

Different methods have been used to examine associative learning in social insects. The Maxilla-Labium Extension Response (MaLER) conditioning paradigm has been used to demonstrate visual associative learning^17–19^ and olfactory learning^20–25^ in ants. Olfactory learning among ants has also been demonstrated using a Y-maze^26–29^ and a free walking arena^30–32^. In addition to visual and olfactory cues, tactile sense can also provide ants with crucial information about the attributes of the surface on which they move, outside and inside the nest. Tactile cues become all the more important for insects in the absence of light^33^. Even though the information obtained through the tactile sense can be important for orientation and navigation in the environment, it has not been investigated as extensively as visual or olfactory senses in terms of its capacity to predict reinforcing stimuli. However, some information on the importance of tactile cues has been gathered in *Cataglyphis fortis* showing that local ground structures can be memorised and recalled by returning foragers in the vicinity of the nest entrance^34^. In *Lasius niger,* however, no evidence of learning of substrate coarseness was found^33^.

Here we used a tropical Ponerine ant, *Diacamma indicum,* as a model for studies on associative learning*. Diacamma indicum* is a 1 cm long black ant found in southern and eastern India, Sri Lanka, Bangladesh and maybe Japan. The colonies are monogynous and monodomous, i.e., comprised of a single reproductive female, termed gamergate, and the colony resides in one nest^35–37^. Nests are typically subterranean^38^. The nest size ranges from 12 to 261 adults, with an average size of 90.2 ± 13.9 adults^38^. *D. indicum* ants have been shown to use natural visual landmarks placed near the nest during relocation^39^. When colonies were visually impaired, they could relocate successfully using tactile cues along the edges of the relocation arena. But these colonies showed increased transportation time and lower tandem running efficiency^40^. When the antennae of *D. indicum* were restricted by attaching the scape of the antennae to the head capsule using non-toxic enamel paint, relocation was successful, but both relocation time and the number of interruptions during tandem running was significantly higher^39^. These findings highlight the importance of tactile and visual modalities for *Diacamma indicum*. In the current study, we explore visual and tactile learning in *Diacamma indicum* at the individual level. As mentioned above, *D. indicum* colonies generally occupy subterranean nests but they are also found in hollow spaces inside bamboo, coconut shells, crevices in rocks, walls and under bricks and logs. In such environments, workers are likely to encounter different surfaces with variable coarseness, such as foliage, soil, bricks and wood. Along with visual cues, tactile cues (surface coarseness) could be used by workers when navigating in these environments.

Associative learning experiments require organisms to be motivated to respond to the reinforcement delivered in learning assays^41^. Relief from a negative stimulus (negative reinforcement), like escape from water^42^ or electric shock^43^, or experience of an aversive stimulus (positive punishment) like quinine solution, has been used as reinforcing stimuli^26^. Petting also serves as motivation in dogs^44–46^. Food has been a quintessential source of reinforcement in many learning paradigms in invertebrates such as bumblebees^47^, honeybees^48–50^ and ants^18,51^. But food motivates animals until they are sated^41^. A longer waiting time between subsequent unloading interactions is experienced by honey bee foragers if the colony is satiated^52^; among ants, receiving a particular type of food^53^ and deprivation of required nutrients^54^ leads to colony satiation. The viscosity of food source (sugar) impacts the number of trips and method through which sucrose solution is shared by nestmates in *D. indicum*^55,56^. *D.* indicum foragers are solitary scavengers that occasionally hunt for termites. They live in small colonies, which contain on average 89.35 ± 38.75 adult females, 0.29 ± 1.19 males and 56.6 ± 42.53 broods of different developmental stages^57^. They hardly store food inside their nests (personal observations), thus being likely to get quickly satiated. Hence, a given individual is unlikely to make several trips to collect food, a condition that would be required for food-related training. To overcome these limitations in examining associative learning, we decided to use a different kind of reinforcing stimulus, namely pupa.

The life cycle of holometabolous insects comprises four developmental stages, namely, egg, larva, pupa, and adult. The first three stages are brood stages that ultimately give rise to the adults^58^. Brood is a prized possession of insect colonies. It is a means to achieve reproductive fitness as it develops into the future reproductive individuals and workforce. Larvae and sometimes pupa can act as reserves of food in insect colonies by exudating nutritious fluid for the adults and they can also be cannibalised as food in times of food scarcity^59–63^. Out of the three stages of brood, the pupae are the most valuable as they need the least investment in rearing before they eclose into adults and become part of the workforce^64,65^. Thus, adults would be selected to take care and protect the pupae and even rescue their pupae if they occasionally get lost. In nature, dozens of species of ants in the genera *Myrmoxenus*, *Chalepoxenus*, *Harpogoxenus*, *Temnothorax,* and *Polyergus* conduct raids to pillage broods from neighboring host colonies and are termed, therefore, as slave-making ants^61,66^. Recently, brood theft has also been documented in *D. indicum*. Workers are involved in opportunistic theft of brood, especially pupae, from a conspecific colony, showcasing the importance of brood for the colony as the thieves put themselves at risk^67–70^.

*D. indicum* foragers collect unguarded pupae whenever available and they steal pupa from relocating colonies, with thieves making several trips to this end (up to 23)^68,69,71^. The discovery of pupae motivated individuals to make several trips to collect them and when unguarded pupae were offered the risk involved for a worker in retrieving it was very low, possibly increasing its motivation. In another study, when workers were challenged with the problem of inserting pupa through a narrow entrance, they again showed high motivation to collect a large number (40.5 ± 2.87) of pupa and tried to insert them into the new nest. Given that pupa can act as a good motivation for making multiple trips^72^, we used it as a reward in our associative learning experiments. Although pupae have been used to motivate ants in different contexts, such as foraging^73^ and navigation^74^, they have not been used in the context of associative learning so far.

We thus asked if individuals of *D. indicum* can learn tactile and visual cues when presented in association with a pupa reward, and if pupae function as a better reward compared to food for learning tactile cues. We then investigated if these ants had established a stronger association between pupa and the tactile or the visual cue when these cues are decoupled during the test phase.

## Materials and methods

### Colony collection

Thirty-five colonies were collected using the nest flooding method^71^ in and around Nadia, West Bengal (22°56′ N, 88°33′ E) between August 2021 through June 2022. Colonies were maintained in the laboratory using a standard methodology^75^ and provided with ad libitum water and ant cake^61,76^. As *D. indicum* individuals are monomorphic, they were examined under a Nikon SMZ-745 Stereomicroscope to search for the gamergate, the sole reproductive female in the colony. Only colonies with a gamergate were used for these experiments. For unique identification, all individuals were marked with non-toxic enamel paints (Testors, Rockford, IL, USA).

### Collection and maintenance of pupae

Pupae were obtained on a rolling basis from freshly collected *D. indicum* colonies. The pupae collected on a day were marked with a specific non-toxic enamel colour to allow identification of when they were collected. We qualitatively observed that ants do not accept pupae older than 8 days post collection. Therefore, pupae that were kept for more than 8 days post collection were not used for these experiments. Between experiments, they were stored in an incubator with 5 to 6 worker ants at 27 °C and 60% relative humidity to ensure they were maintained in a viable condition.

### Experimental setup and protocol

**Figure 1:**
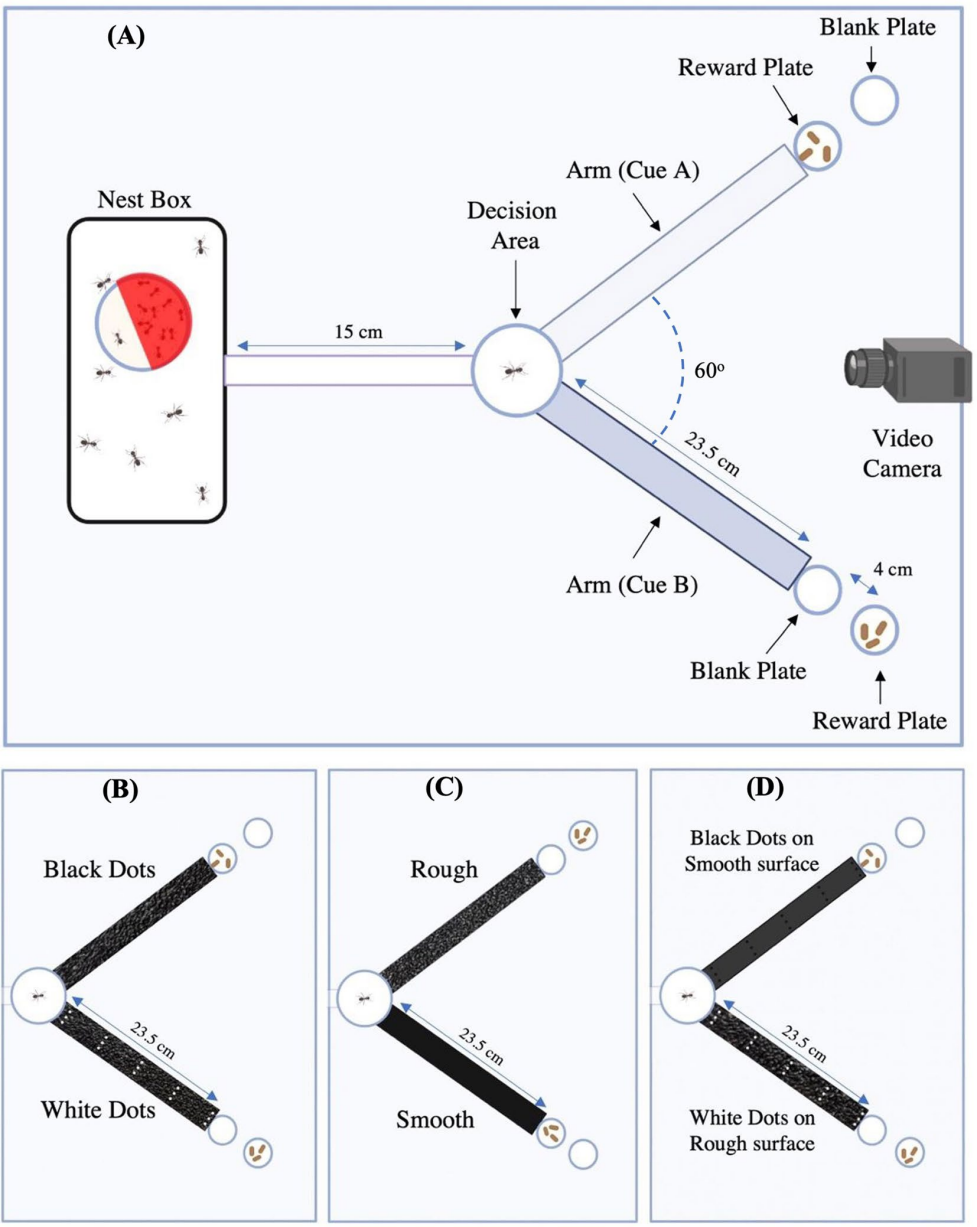
Setup for associative learning. (**A**) Schematic of the Y-maze (the reward plate contains either food or pupae), (**B**) Visual Learning setup with arms having white dots on a rough surface and black dots on a rough surface, (**C**) Tactile Learning setup with arms having rough and smooth surfaces, (**D**) Visual and Tactile Cue Conflict setup with arms having white dots on rough surface and black dots on smooth surface (created in Biorender.com).

Colonies consisted of 91.9 ± 36.8 adult females. All experiments were carried out in a 60 × 90 cm arena made of plexiglass with walls coated with Vaseline^®^ jelly to prevent ants from escaping. The intensity of light in the arena was between 100 and 130 lux, measured using 101A Luxmeter (HTC Instruments, India). Low light intensities were preferred to minimise any reflection from surfaces that were coated with acrylic paints (Fevicryl^®^ Pidilite, India). The coat of paint on the surfaces was allowed to dry for 48 h before they were used for any experiments. The experiments were carried out at a temperature of 25 ± 2 °C. The setup (Fig. 1) consisted of a nest box of size 21 × 15.5 × 8.5 cm with an opening (2 × 2 cm) at the bottom center of one of the walls. The opening was connected to a Y-maze. On emerging from the nest box, ants encounter a path made of plastic (15 cm) leading to a circular junction termed the “decision area” (9 cm diameter). The path bifurcated from the decision area into arms (23.5 cm long) made of plastic and each of them led to two small Petri-plates, one in contact with the arm and the other separated by 4 cm. The arena was filled with water to a depth of 0.5 cm to prevent ants from leaving the path. A maximum of 5 worker ants from a colony were trained and tested, and each ant was used only once. After testing, an individual was permanently separated from the colony. Every experiment was divided into two phases—a training and a test phase.

### Training phase

The objective of the training phase is to allow ants to associate a cue with the reward. The conditioned stimuli were either visual cues or tactile cues or a combination of both. Pupa was the unconditioned stimulus. In a given trial, the two arms had two different cues. At the end of each arm, two Petri plates were placed—one in contact and one separated from the arm. The arm that carried the cue to be learned had pupae accessible in the petri-plate. The other arm (non-rewarded) also had pupae in the petri-plate but it was inaccessible to ants because of the separation between the plate and the arm. This was done to balance olfactory cues on both arms. The pupae used during the experiment could be from the colony of the individual being trained, a foreign colony, or both. A colony was kept in the nest box and the ants were allowed to explore the path. The first ant to discover the pupae and return with one to the decision area was chosen for training and was selectively allowed to make successive training trips. All other ants entering the decision area were gently placed back in the nest box using feather forceps. There was no fixed intertrial interval, the ant undergoing training was allowed to move into the two arms freely and return to the nest with reward at its convenience. Every ant underwent 10 training runs. After each run, the arms were cleaned with a microfiber cloth soaked in 40% ethanol to remove any pheromone or cuticular hydrocarbons that may have collected on them, and the position of the arms was interchanged randomly to inhibit the individual from developing directional memory of the reward. After each run, a pupa was removed from the other side to avoid an imbalance of olfactory cues, if any. Thus, at any given time, both arms had an equal number of pupae.

### Test phase

The test phase consisted of a single un-reinforced test run conducted as soon as the focal trained ant exited the nest and came to the decision point after the 10 training runs. In the test run, no pupae were placed at the end of either arm. The position of the conditioned cue during the test run was randomised in a similar manner as the training sessions. The test run ended when the worker left the decision area and returned to the nest box for the first time. During the test run, the following data were recorded for each individual:

1. Decision—when a trained ant walked from the nest to the decision point, entered one of the arms and touched the reward plate, a decision in the favour of the cues on that particular arm was recorded. This decision would be scored as the correct decision if the focal ant touches the reward plate at the end of the arm that holds the conditioned cue. On the other hand, if the focal ant touches the reward plate at the end of the arm that holds the non-conditioned cue, it would be scored as an incorrect decision. Note that, in the tactile and visual cue conflict experiment, there was no correct or incorrect decision.
2. Time spent—after an ant left the decision area and walked into any of the arms, we measured the time it spent within each arm separately. We stopped measuring time when it returned back to the nest. The sum of the time spent in both arms is designated as the total time spent by the test ant. Note that the test ant can make multiple trips to each of the arms before finally returning back to the nest. From the data obtained, we calculated the proportion of time spent in each of the arms by dividing the time spent in a particular arm by the total time spent. This normalisation is performed because the duration of the test run was highly variable among individuals. The proportion of time spent in the arms with different cues was compared. If an ant spent significantly more time in an arm with the conditioned cue during the test, we concluded that the focal ant had learned to associate that cue with the reward^47,77^.

### Tactile associative learning

In order to determine if ants learn to associate a tactile cue with a reward, one arm of the Y-maze had 80-Grit sandpaper painted black (rough), and the other had the reverse side of the 80-Grit sandpaper painted black (smooth). Using the reverse side of the same sandpaper allowed us to keep the amount of material equal on both arms to provide conditions in which all the other cues were comparable. We trained 24 individuals from 8 different colonies to associate either cue with pupa reward—i.e. half of the individuals got pupa reward after travelling on the rough surface, while the other half got a pupa reward after travelling on the smooth surface. The training phase was followed by a test run, as mentioned in the previous section.

We performed a separate set of experiments to determine whether ants associate a tactile cue with food instead of pupa as a reward. Food consisted of 33.33% sucrose (Sucrose Pure, Merck, Darmstadt, Germany) solution as used in other experiments with ants^17^. While the other non-conditioned arm contained a blank reward plate. Colonies were starved for three days to increase their motivation to forage similar to what had been done previously^78^. Twelve individuals from 3 different colonies were trained with food as the unconditioned stimulus. The tactile cue was the conditioned cue and half of the ants were trained to associate the rough surface with food reward while the remaining half was trained to associate the smooth surface with a food reward. All other procedures of this experiment were identical to that offering the same tactile cues and pupa as reward.

Using another set of experiment, we evaluated, the association of tactile cues among visually impaired ants. By visually impairing them, we ensured that these ants did not have any exposure to the visual components of the Y maze setup. All components of this experiment were identical to the tactile associative learning experiment and used pupae as the unconditional stimulus. We trained 15 visually impaired ants from 5 colonies for this experiment (see supplementary materials section for detailed methodology).

### Visual associative learning

In order to determine if ants learn to associate visual cues with pupa as reward, both arms of Y-maze were overlaid with 80-Grit sandpaper that was painted black using acrylic paint and on one arm presented triplets of white dots (~ 2 mm in diameter) placed at 3.1 cm intervals. On the other arm, triplets of black dots of the same size were marked at the same distance intervals to balance any change in texture the dots may have introduced. Thus, ants had to learn to discriminate the arms based on the presence/absence of the white dots. We trained 24 individuals from 9 different colonies to associate either cue with pupa—i.e. half of the individuals got rewarded after travelling on the arm with white dots on the black background, while the other half got rewarded after travelling on the arm with black dots on the black background. The training phase was followed by a test run conducted, as mentioned in the previous section.

### Visual and tactile cue conflict

In order to determine if ants show any preference between the visual and the tactile cues, we performed two experiments. In the first case, one arm had a black painted 80-Grit sandpaper (rough) overlaid with white dots while the other arm had the reverse side of the same sandpaper painted black (smooth) overlaid with black dots (similar to the visual associative learning experiment). We trained 24 ants from 10 colonies to associate the combination of rough surface with white dots with pupa as a reward using the methodologies described before. During the test, we created a conflict between the learned cues as one arm exhibited the black-painted sandpaper (rough) with black dots, and the other a black-painted smooth surface with white dots.

In the second experiment, one arm presented a black-painted reverse side of the sandpaper (smooth) overlaid with white dots while the other arm presented the black-painted rough side of the sandpaper overlaid with black dots. In the test we created again a conflict between learned cues as one arm presented the black painted rough sandpaper with white dots while the other arm presented the smooth reverse side of the sandpaper with black dots. Twenty-four ants from 8 colonies were trained to associate the combination of smooth surface with white dots with the pupa reward using the methodology already described. Thus, in the two experiments, the training phase had a visual and tactile cue in association with the reward, but in the test phase, the cues were decoupled between the arms, i.e., the visual cue on one arm was combined with the tactile cue on the opposite arm and vice versa, to determine if ants had a preference between visual and tactile cues. The test run was performed in a similar manner as described in the previous section. In the test, if ants spent significantly more time in the arm with the visual or the tactile cue that was previously rewarded, we concluded that a preference to associate that cue with the reward existed.

### Measurement of surface textures

**Figure 2:**
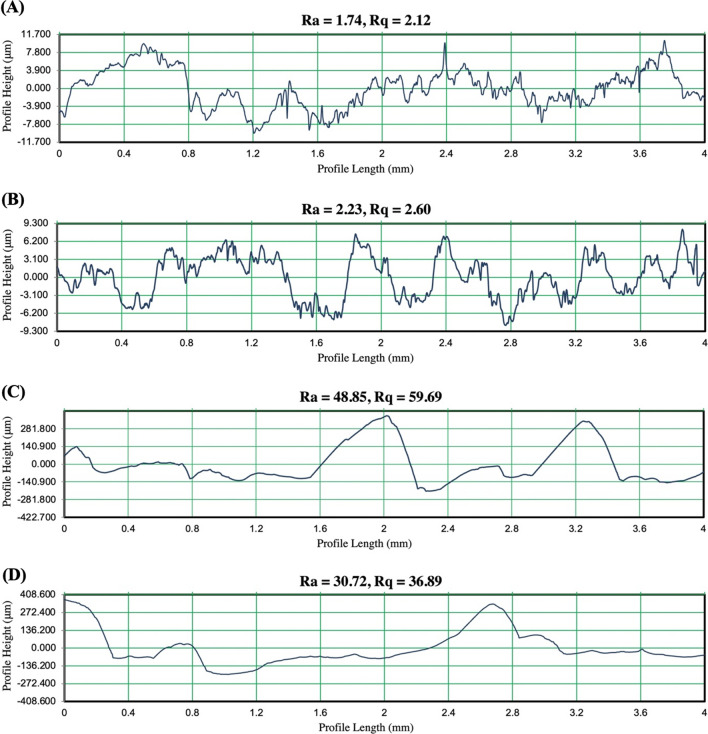
Two-dimensional profiles of the surfaces used in the experiments. The graphs represent the profiles of (**A**) smooth painted surface, (**B**) dot on a smooth painted surface, (**C**) Rough (80 Grit sandpaper) painted surface, (**D**) dot on rough painted surface.

Two-Dimensional topographies of the surfaces used were measured using Zeiss SURFCOM TOUCH 50 surface profilometer with JIS2001/2013 evaluation standards over an evaluation length of 4 mm and a cut-off value of 0.8 mm (Fig. 2). Roughness was characterised using *Ra* and *Rq* values. *Ra* measures the arithmetic mean of the absolute deviations of the surface from the mean line over a sampling length. In other words, *Ra* is the average of individual measurements of valleys and peaks along a surface. *Rq,* also called the root-mean-square (RMS), is the standard deviation surface heights distribution. *Rq* is more sensitive to large deviations from the mean line^79^.

### Statistical analysis

Decisions made during the test phase were analysed using a Binomial test considering 0.5 expected probability. The remaining data were analysed using the “MASS” package^80^ to perform Generalized Linear Model (GLM) with a negative binomial distribution, “stats” base package to perform Linear Model (LM), and “betareg” package^81^ for the regression model with beta distribution. Before modelling, the Shapiro test was performed to assess normality and the “fitdistrplus” package^82^ was used to assess the error distribution of the response variables. For all statistical tests, the significance was set at p < 0.05. Unless mentioned otherwise, mean ± standard deviation (S.D.) values are provided. All the statistical tests were performed using R (version 4.2.1, R Core Team, 2022) and the graphical representations were prepared using PAST (version 4.03)^83^ and Microsoft Excel, 2019.

## Results

### Tactile associative learning with pupae as reward

**Figure 3:**
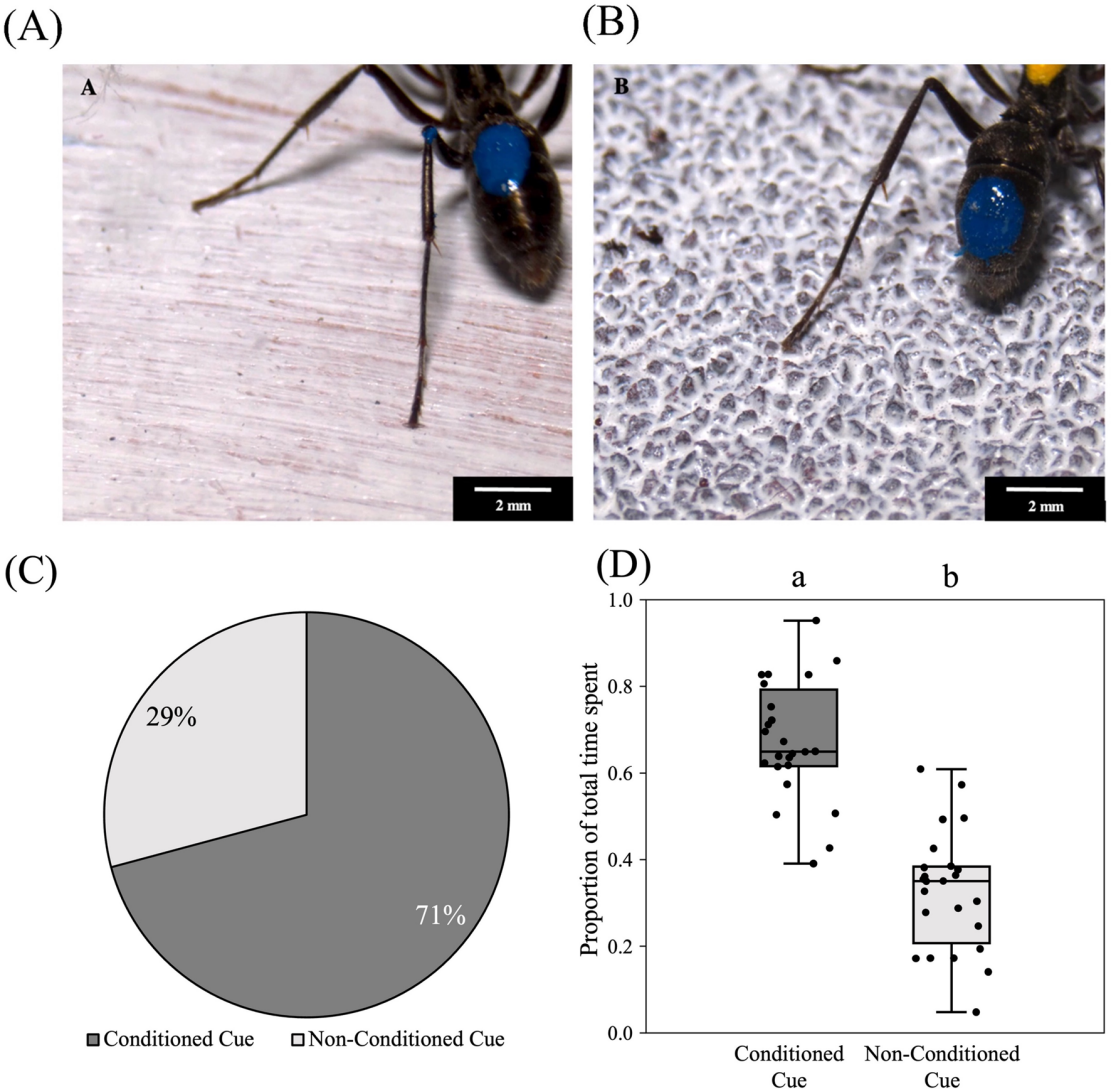
Tactile association experiment with Pupa as a reward: different cues used for conditioning—smooth surface (**A**) and rough surface (80 Grit Sandpaper) (**B**), with an ant walking on it. Note that for the purpose of these pictures the surfaces have been painted white. In the experiment, they were painted black. (**C**) percentage ants took decisions in favour of conditoned and non-conditioned cue during test run. (**D**) The proportion of time spent on the two cues during the test has been plotted using jittered box plots (N = 24). An equal number of ants were conditioned on the rough and smooth tactile cues. The bold black horizontal line inside the boxes represents the median, the box represents the interquartile range (IQR), and the whiskers of the boxes represent the data points which are within 1.5 × IQR. Different letters on the boxplots represent a significant difference (p < 0.05) between the two categories.

Out of 24 ants trained, 17 chose the conditioned cue during the test run [70.8 ± 18.2% (95% confidence interval); Binomial test: N = 24, k = 17, *p* = 0.031]. Ants spent a significantly greater proportion of time in the arm with the conditioned cue (0.672 ± 0.137) than in the arm with the non-conditioned cue (0.327 ± 0.137) (Beta Regression: *est.* = − 1.45. *z* = − 7.95, *p* < 0.001) (Fig. 3A–D; Table S1). Ants can thus learn tactile cues based on their association with pupa as positive reinforcement.

An additional set of experiments with visually impaired ants, also indicates the same results. Out of 15 ants trained, 12 chose the conditioned cue during the test run (Binomial test: N = 15, k = 12, *p* = 0.019). Further, ants spent a significantly greater proportion of time on the arm with the conditioned cue (0.773 ± 0.157) than on the arm with the non-conditioned cue (0.226 ± 0.157) (Beta regression: *est.* = − 2.42,  z = − 8.44, *p* < 0.001) (Fig. S1).

### Tactile associative learning with food as reward

**Figure 4:**
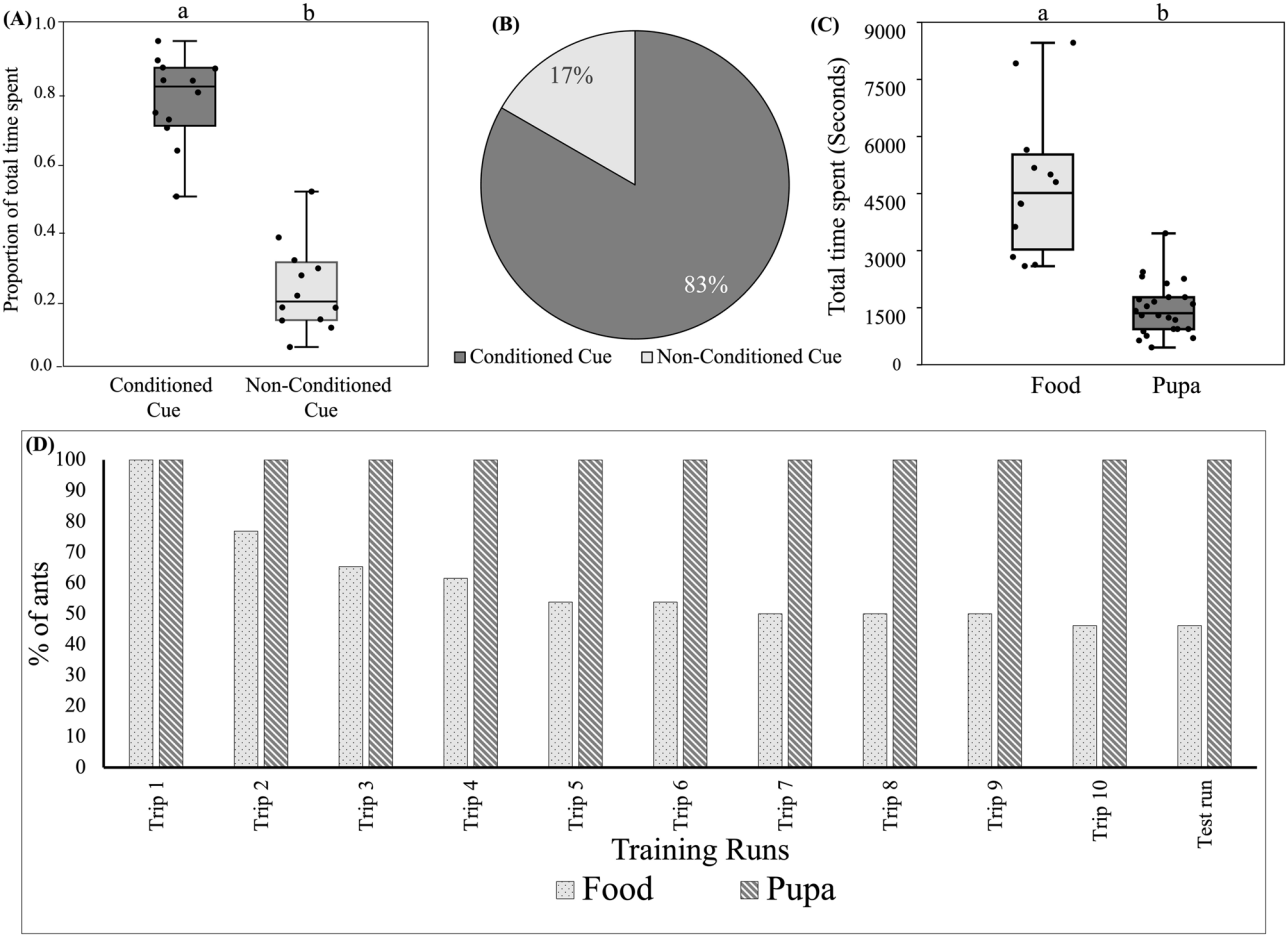
Tactile associative learning with food as a reward: (**A**) The proportion of time spent on the two cues during the test has been plotted using jittered box plots (N = 12). An equal number of ants were conditioned on the rough and smooth tactile cues. (**B**) Percentage ants took decisions in favour of conditoned and non-conditioned cue during test run. (**C**) Total time spent from first training run to test run by ants for food (N = 12) and pupal reward (N = 24). (**D**) Attrition rate for pupa and food rewards are being compared by plotting the number of ants (%) that successfully performed consecutive training runs for both the rewards.

Out of 12 ants trained, 10 chose the conditioned cue during the test run [83.3 ± 27.1% (95% confidence interval); Binomial test: N = 12, K = 10, *p* = 0.021]. Ants spent a significantly greater proportion of time in the arm with the conditioned cue (0.773 ± 0.127) than in the arm with the non-conditioned cue (0.226 ± 0.127) (Beta Regression: *est.* = − 2.44, *z* = − 8.97, *p* < 0.001) (Fig. 4A and B; Table S1). Thus, tactile cues can also be learned based on their association with sucrose solution.

### Comparison of tactile learning using food and pupae as rewards

To determine the reinforcing strength of food and pupa rewards, we conducted three different analyses. We first compared the proportion of “correct” choices (decision in favour of conditioned cue) taken by ants in the test run for both food and pupa rewards, where the result showed no significant difference in the proportion of “correct” choices (z = 0.879, *p* = 0.379). Next, we compared the total time taken by ants from the first training run to the test run, that is 11 runs for both scenarios. Ants took significantly more time when food was provided as reward (4904.66 ± 1895.43 s) as compared to pupae as reward (1615 ± 697.90 s) (Generalized Linear Model: *est.* = − 1.11, *z* = − 7.94, *p* < 0.001) (Fig. 4C; Table S1) to complete training and test. Next, we compared the proportion of time spent in the conditioned arm in the test phase for both cases; ants spent a significantly higher proportion of time in the conditioned arm when the reward was food (0.773 ± 0.137) as compared to pupae (0.672 ± 0.127) (Beta Regression: *est.* = − 0.47, *z* = − 2.08, *p* = 0.037) (Table S1). Additionally, we examined the rate of attrition for both food and pupae rewards (i.e. “the number of ants that did not return to the maze for the subsequent training run”), by calculating the percentage of ants that successfully performed in the consecutive runs from the first training run to the test run. None of the ants failed to complete the 10 training runs and the test run when pupae were provided as a reward. On the other hand, only 46% of ants were able to complete the test run when the food was provided as a reward (Fig. 4D, Table S1).

### Visual associative learning

**Figure 5:**
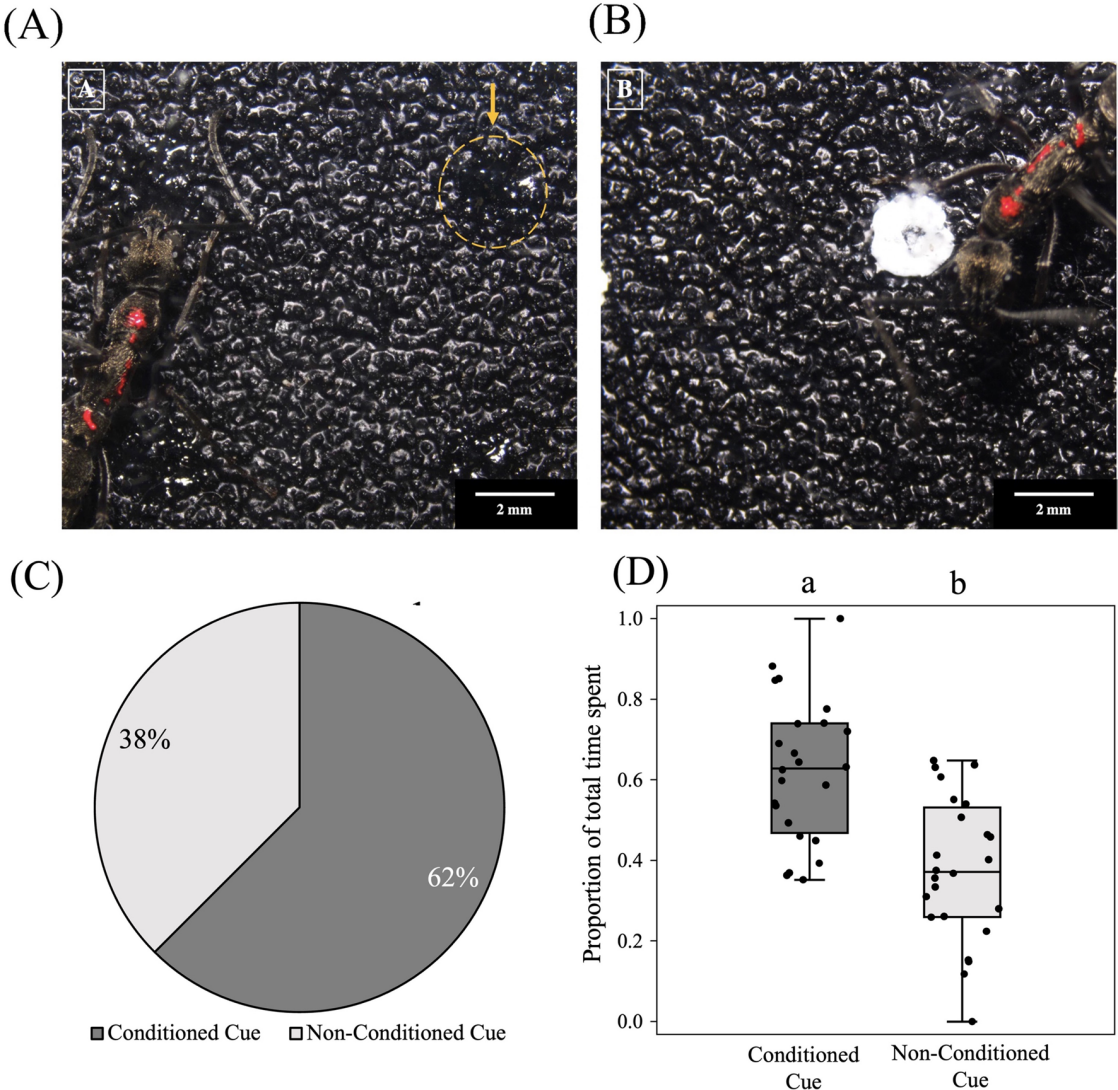
Visual association experiment: different cues used for conditioning—black dots on a rough surface (**A**) (arrow marks a black dot), and white dots on a rough surface (**B**), with an ant walking on it. (**C**) Percentage ants took decisions in favour of conditioned and non-conditioned cues during the test run. (**D**) The proportion of time spent on the two cues during the test has been plotted using jittered box plots (N = 24). An equal number of ants were conditioned on the white and black visual cues. The bold black horizontal line inside the boxes represents the median, the box represents the interquartile range (IQR), and the whiskers of the boxes represent the data points which are within 1.5 × IQR. Different letters on the boxplots represent a significant difference (p < 0.05) between the two categories.

Out of the 24 ants trained, 15 chose the conditioned arm during the test run [62.5 ± 19.3% (95% confidence interval); Binomial test: N = 24, k = 15, *p* = 0.153]. Despite this lack of significant choice for the conditioned arm at the first exploration on the test run, ants spent a significantly greater proportion of time in the arm with the conditioned cue (0.623 ± 0.177) compared to the arm with the non-conditioned cue (0.377 ± 0.177) (Linear Regression: *est.* = − 0.24. *t* = − 4.7, *p* < 0.001) (Fig. 5A–D; Table S1).

### Tactile and visual cue conflict

**Figure 6:**
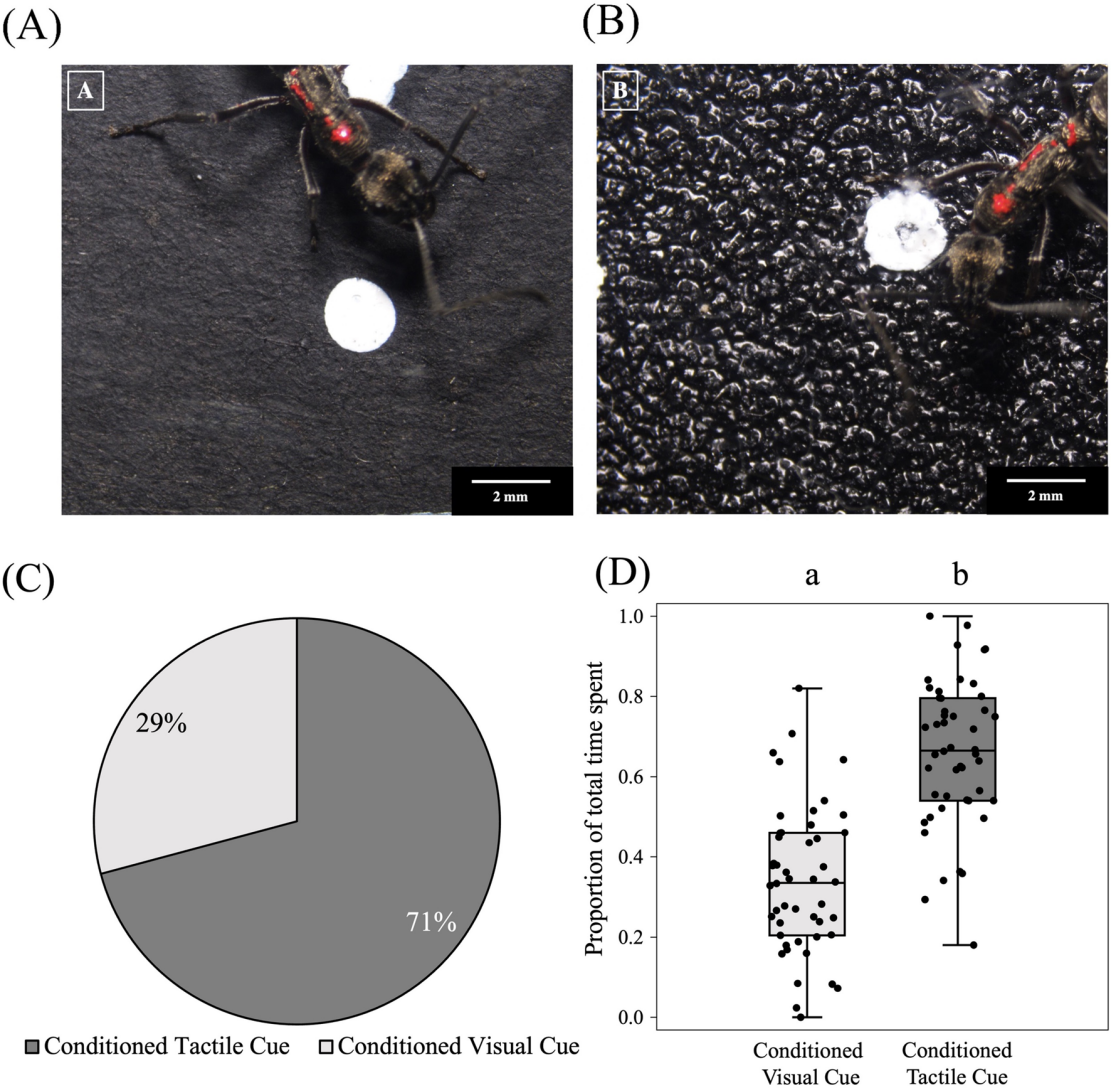
Visual and tactile cue conflict experiment: different cue combinations used—white dots on black rough surface (**A**) and white dots on black smooth surface (**B**), have been shown with an ant walking on it. (**C**) Percentage ants took decisions in favour of conditoned and non-conditioned cue during test run. (**D**) The proportion of total time spent between conditioned visual and conditioned tactile cues has been plotted using jittered box plots (N = 48). The bold black horizontal line inside the boxes represents the median, the box represents the interquartile range (IQR), and the whiskers of the boxes represent the data points which are within 1.5 × IQR. Different letters on the boxplots represent a significant difference (p < 0.05) between the two categories.

In the next step, we explored if ants preferred to associate pupa with visual or tactile cues when they are decoupled from each other during the test as compared to the training. In this conflict test situation, out of 48 ants trained, 34 chose based on the tactile cue during the test [70.8 ± 12.8% (95% confidence interval); Binomial test: N = 48, k = 34, *p* = 0.002]. Ants spent a significantly greater proportion of time in contact with the tactile cue (0.660 ± 0.181) as compared to the arm displaying the visual cue (0.339 ± 0.181) (Linear Regression: *est.* = − 0.32, *t* = − 8.6, *p* < 0.001) (Fig. 6A–D; Table S1).

## Discussion

In this study, we investigated associative learning of visual and tactile cues in *D. indicum* using a Y-maze-based conditioning paradigm and pupae as reward. For testing tactile association, we used sandpapers of different grain size, a procedure adapted from Seidl and Wehner^34^ who reported tactile differentiation of surfaces with different coarseness in the context of nest-homing behaviour in *Cataglyphis fortis*. Tactile learning was also observed in *Ectatomma ruidum* using a MaLER response paradigm, wherein the response was amplified when the tactile stimulus (contact of a paper strip with the antenna) was combined with a chemical/olfactory stimulus^19^. However, in *Lasius niger*, individual ants failed to associate sands of different coarseness with a reward, although their locomotory speed and trajectories were affected by surface properties^33^. In the current experiments, we found that *D. indicum* ants learn to associate smooth and rough surfaces with pupa reward. The number of ants choosing the conditioned cue and the proportion of time spent on it are both significantly different from a random choice. It is possible that these ants perceive the coarseness of the ground as a visual cue rather than a tactile cue although this possibility is quite unlikely given the relatively poor resolution of the insects’ eye. In order to be sure, we did additional experiments with vision impaired ants, i.e., ants whose compound eye was occulated with paint fully thus blocking any visual inputs. Vision impaired ants also showed a clear association between pupa reward and tactile cues (see supplementary material: Tactile associative learning with visually impaired ants’ experiment).

Previous studies on insect learning mostly dealt with appetitive and aversive learning and these approaches adequately help to shed light on neural functionality in relation to learning and memory formation^2,84^. Appetitive learning has been mostly studied using food reward, usually sucrose solution. Colonies that receive a particular type of food show satiation for that type of food^53^. Ants also altered their feeding preference when they were deprived of their required nutrients in both the starvation and satiation scenarios^52,54^. In the case of *D. indicum* we had observed that foragers make only a couple of trips within a short time when they received food rewards, which represents a difficulty for learning assays based on this kind of reward as several assays are typically needed to evaluate the presence of learning and memory. This was the reason for using pupa as reward. In the next step, we compared the associative performance of ants that experienced either food or pupae as reward. On conducting associative learning experiments with tactile cues paired with food reward, we checked the time taken to complete the training involving 10 runs with food and also the attrition for food. Ants were able to learn to associate tactile cues with food reward but the time taken to do so was significantly longer than in the case of pupae rewards (approximately 3 times as long). Remarkably, there was zero attrition in the case of pupae reward. In the case of food rewards, a significantly small proportion (46%) of ants that started the training finished it. This highlights the higher motivation provided by pupae as compared to food rewards.

Pupae can certainly be used as reward for associative studies in the case of ants that perform brood raids, but it can also be used for most other ant species where adults are selected to care and rescue their pupae as this is the most invested brood item in the colony. Only experiments with multiple other species of ants in different contexts can confirm this hypothesis. In addition, given that appetitive rewards such as food trigger specific biogenic-amine signalling in the brain of social insects in the context of learning experiments^85^, it would be interesting if pupa triggers the same kind of signalling as food in the ants’ brain. To summarize, by utilising pupae as a reward we were able to overcome some limitations imposed by conventional rewards like food in this solitary scavenging species of ant living in small colonies, while preserving the ecological relevance of the research. To the best of our knowledge, there have been no reports of the use of developing brood in the form of pupae as a reinforcement for associative learning.

In the present study we found that individual ants can learn to differentiate maze arms using visual cues displayed on the ground within 10 training runs if the total time spent on the conditioned arm was considered as proxy of their learning success. On the contrary, focusing on the initial decision did not report a significant choice of the arm displaying the conditioned visual cues. It is possible that these ants were not able to perceive the pattern of white dots on a black surface until they came very close to it given the small size of the dots and the poor spatial resolution of the insect eyes, resulting therefore in erroneous initial decisions. If the patterns used were larger and presented either laterally to induce optic flow or frontally^51^ to allow better discrimination, this limitation even in terms of the initial choice could possibly be overcome. In another scavenger ant, *Cataglyphis fortis*, visual learning of landmarks, much bigger in size (7 cm), on the ground in the context of nest homing has been observed^34^. At the level of a colony, *Myrmica ruginodis* can distinguish between specific shapes and their orientations in the context of foraging^86^. The specificity of visual cue association can be explored further by training ants with different patterns, sizes, shapes and colours in future experiments. Understanding the visual acuity of these ants will help us design and provide the appropriate visual cues.

Cue conflicts can arise in nature where information from different sensory modalities coexist, and when one or more sensory cues vary in consistency and reliability^87^. Using visual and mechanical tactile stimuli, this study investigates the preference of *D. indicum* ants between the cues after exposing them to both cues concurrently. When both tactile and visual cues are provided during the training, and then placed in conflict during the test, ants showed a significant preference for the tactile cue. Both the initial decisions and the proportions of time spent were in favour of the tactile cues. It is possible that this preference is due to the low salience of visual cues discussed above, which may have rendered tactile cues more reliable. The preference may be altered if visual cues are made more prominent. There have been earlier studies in cue conflict between—olfactory and route memory in *Lasius niger*^27^, side bias and trail pheromone in *Acromyrmex subterraneus*^88^ and celestial and terrestrial cue in *Melophorus bagoti*^89^. In *Ectatomma ruidum,* difference in strength of chemotactile and visual learning were studied^19^. However, the (chemo)tactile cue used was not purely mechanical, and these cues were not presented in a concurrent manner to the ants but different groups of ants were exposed to one or the other stimuli and compared.

In conclusion, we demonstrate that *Diacamma indicum* ants learn to associate tactile and visual cues with a pupal reward and spent in consequence significantly more time on the arm displaying the conditioned cue. In the case of the current set of cues, they showed a clear preference for tactile cues over visual cues when these two cues were decoupled from each other. Our study sheds light on the importance of studying tactile cues and their role in decision-making by ants and adds to the relatively sparse literature on tactile associative learning in ants. Our conditioning paradigm presents a novel kind of reinforcement, pupa, inducing long-lasting motivational search opening up new possibilities for conducting experiments requiring extended training. It will be interesting to investigate the sensitivity and selectivity to different tactile and visual cues that are derived from the sensory dimension of the organism and understand when and how fast these associations can be achieved and if the proximate mechanisms by which these associations function are similar.

### Supplementary Information

Supplementary Information 1.

Supplementary Information 2.

## Data Availability

All data analysed during this study are included in supplementary information files.
