# Supplementary material for: Using pupae as appetitive reinforcement to study visual and tactile associative learning in the Ponerine ant Diacamma indicum
Source: Sci Rep. 2023 Sep 20;13:15609. doi: 10.1038/s41598-023-42439-w (PMC10511714; doi:10.1038/s41598-023-42439-w)
Supplement: Supplementary file 1 — Supplementary Information 1. [file 41598_2023_42439_MOESM1_ESM.pdf]

R Markdown for Visual and Tactile Associative Learning

Install Packages

```
library(betareg)
library(MASS)
```

Tactile Associative Learning With Food As A Reward

```
Mod_Tactile_Food<-betareg(Proportion.of.Time.spent~Arm,data = Tactile_Learnin
g_Food)
summary(Mod_Tactile_Food)

##
## Call:
## betareg(formula = Proportion.of.Time.spent ~ Arm, data = Tactile_Learning_
## Food)
##
## Standardized weighted residuals 2:
##     Min      1Q  Median      3Q     Max
## -2.0175 -0.6773  0.0000  0.6773  2.0175
##
## Coefficients (mean model with logit link):
##                          Estimate Std. Error z value Pr(>|z|)
## (Intercept)                1.2225     0.1891   6.464 1.02e-10 ***
## ArmNon-Conditioned Cue   -2.4451     0.2725  -8.974  < 2e-16 ***
##
## Phi coefficients (precision model with identity link):
##       Estimate Std. Error z value Pr(>|z|)
## (phi)   11.719      3.305   3.546 0.000391 ***
## ---
## Signif. codes:  0 '***' 0.001 '**' 0.01 '*' 0.05 '.' 0.1 ' ' 1
##
## Type of estimator: ML (maximum likelihood)
## Log-likelihood: 18.81 on 3 Df
## Pseudo R-squared: 0.7749
## Number of iterations: 16 (BFGS) + 2 (Fisher scoring)
```

Tactile Associative Learning With Pupa As A Reward

```
Mod_Tactile_Pupa<-betareg(Proportion.of.Time~Arm,data = Tactile_Learning_Pup)
summary(Mod_Tactile_Pupa)

##
## Call:
## betareg(formula = Proportion.of.Time ~ Arm, data = Tactile_Learning_Pupa)
##
## Standardized weighted residuals 2:
```

```
##     Min      1Q  Median      3Q     Max 
## -3.1313 -0.4790  0.0000  0.4790  3.1313 
## 
## Coefficients (mean model with logit link):
##                         Estimate Std. Error z value Pr(>|z|)    
## (Intercept)               0.7278     0.1286   5.661 1.50e-08 ***
## ArmNon-Conditioned Arm  -1.4555     0.1831  -7.950 1.87e-15 ***
## 
## Phi coefficients (precision model with identity link):
##       Estimate Std. Error z value Pr(>|z|)    
## (phi)   10.256      2.012   5.097 3.45e-07 ***
## ---
## Signif. codes:  0 '***' 0.001 '**' 0.01 '*' 0.05 '.' 0.1 ' ' 1 
## 
## Type of estimator: ML (maximum likelihood)
## Log-likelihood: 27.72 on 3 Df
## Pseudo R-squared: 0.5518
## Number of iterations: 14 (BFGS) + 2 (Fisher scoring)
```

Tactile Associative Learning With Food and Pupa Reward (Time spent on arms)

```
Mod_Tactile_Pupa_Food<-betareg(Proportion.of.time.spent~Reward,data = Food.vs
.pupa_Time.Spent)
summary(Mod_Tactile_Pupa_Food)
```

```
## 
## Call:
## betareg(formula = Proportion.of.time.spent ~ Reward, 
##     data = Food.vs.pupa_Time.Spent)
## 
## Standardized weighted residuals 2:
##     Min      1Q  Median      3Q     Max 
## -1.8456 -0.5230 -0.2067  0.6447  3.2034 
## 
## Coefficients (mean model with logit link):
##             Estimate Std. Error z value Pr(>|z|)    
## (Intercept)   1.2101     0.1949   6.210 5.31e-10 ***
## RewardPupa   -0.4792     0.2300  -2.083   0.0372 *  
## 
## Phi coefficients (precision model with identity link):
##       Estimate Std. Error z value Pr(>|z|)    
## (phi)   10.692      2.436   4.389 1.14e-05 ***
## ---
## Signif. codes:  0 '***' 0.001 '**' 0.01 '*' 0.05 '.' 0.1 ' ' 1 
## 
## Type of estimator: ML (maximum likelihood)
## Log-likelihood: 23.23 on 3 Df
## Pseudo R-squared: 0.113
## Number of iterations: 16 (BFGS) + 2 (Fisher scoring)
```

Tactile Associative Learning With Food and Pupa Reward (Total Time Spent)

```
Mod_Tactile_PF_Total_Time<-glm.nb(Total.Time~ Reward,data = Food.vs.pupa_Tota
l.Time.Spent)
summary(Mod_Tactile_PF_Total_Time)
```

```
##
## Call:
## glm.nb(formula = Total.Time ~ Reward,
##     data = Food.vs.pupa_Total.Time.Spent, init.theta = 6.409473488, link =
## log)
##
## Deviance Residuals:
##     Min       1Q   Median       3Q      Max
## -2.1470  -0.9525  -0.1843   0.4360   2.3373
##
## Coefficients:
##                             Estimate Std. Error z value Pr(>|z|)
## (Intercept)                   8.4979     0.1141  74.479  < 2e-16 ***
## RewardPupa  -1.1109     0.1398  -7.946 1.93e-15 ***
## ---
## Signif. codes:  0 '***' 0.001 '**' 0.01 '*' 0.05 '.' 0.1 ' ' 1
##
## (Dispersion parameter for Negative Binomial(6.4095) family taken to be 1)
##
##     Null deviance: 105.024  on 35  degrees of freedom
## Residual deviance:  36.934  on 34  degrees of freedom
## AIC: 596.03
##
## Number of Fisher Scoring iterations: 1
##
##
##               Theta:  6.41
##           Std. Err.:  1.48
##
##  2 x log-likelihood:  -590.033
```

Tactile Associative Learning With Visually Impaired Ants

```
Mod_Tactile_VI<-betareg(Proportion.of.time.spent~Arm,data = Tactile.Learning_
Blinds)
summary(Mod_Tactile_VI)
```

```
##
## Call:
## betareg(formula = Proportion.of.time.spent ~ Arm, data = Tactile.Learning_
## Blinds)
##
## Standardized weighted residuals 2:
##     Min      1Q  Median      3Q     Max
## -1.8673 -0.7299  0.0000  0.7299  1.8673
##
```

```
## Coefficients (mean model with logit link):
##                          Estimate Std. Error z value Pr(>|z|)    
## (Intercept)                1.2109     0.1976   6.129 8.86e-10 ***
## ArmNon-Conditioned cue    -2.4217     0.2868  -8.443  < 2e-16 ***
## 
## Phi coefficients (precision model with identity link):
##       Estimate Std. Error z value Pr(>|z|)    
## (phi)    8.084      2.025   3.992 6.56e-05 ***
## ---
## Signif. codes:  0 '***' 0.001 '**' 0.01 '*' 0.05 '.' 0.1 ' ' 1 
## 
## Type of estimator: ML (maximum likelihood)
## Log-likelihood: 19.41 on 3 Df
## Pseudo R-squared: 0.7278
## Number of iterations: 14 (BFGS) + 2 (Fisher scoring)
```

Visual Associative Learning

```
Mod_Visual<-lm(Proportion.of.Time~Arm,data = Visual.associative.Learning)
summary(Mod_Visual)
```

```
## 
## Call:
## lm(formula = Proportion.of.Time ~ Arm, data = Visual.associative.Learning)
## 
## Residuals:
##     Min      1Q  Median      3Q     Max 
## -0.3769 -0.1209  0.0000  0.1209  0.3769 
## 
## Coefficients:
##                          Estimate Std. Error t value Pr(>|t|)    
## (Intercept)               0.62313    0.03628  17.174  < 2e-16 ***
## ArmNon-Conditioned Arm -0.24625    0.05131  -4.799 1.72e-05 ***
## ---
## Signif. codes:  0 '***' 0.001 '**' 0.01 '*' 0.05 '.' 0.1 ' ' 1
## 
## Residual standard error: 0.1777 on 46 degrees of freedom
## Multiple R-squared:  0.3336, Adjusted R-squared:  0.3191 
## F-statistic: 23.03 on 1 and 46 DF,  p-value: 1.72e-05
```

Visual and Tactile cue conflict

```
Mod_VT<-lm(Proportion.of.time.spent~ Arm,data = Visual.and.Tactile.Cue.Confli
ct)
summary(Mod_VT)
```

```
## 
## Call:
## lm(formula = Proportion.of.time.spent ~ Arm, data = Visual.and.Tactile.Cue
##.Conflict)
## 
## Residuals:
```

```
##     Min      1Q  Median      3Q     Max
## -0.4801 -0.1201  0.0000  0.1201  0.4801
##
## Coefficients:
##                             Estimate Std. Error t value Pr(>|t|)
## (Intercept)                  0.66010    0.02626  25.140  < 2e-16 ***
## ArmConditioned Visual Cue -0.32021    0.03713  -8.623  1.55e-13 ***
## ---
## Signif. codes:  0 '***' 0.001 '**' 0.01 '*' 0.05 '.' 0.1 ' ' 1
##
## Residual standard error: 0.1819 on 94 degrees of freedom
## Multiple R-squared:  0.4417, Adjusted R-squared:  0.4357
## F-statistic: 74.36 on 1 and 94 DF,  p-value: 1.549e-13
```
