# Supplementary material for: Using pupae as appetitive reinforcement to study visual and tactile associative learning in the Ponerine ant Diacamma indicum
Source: Sci Rep. 2023 Sep 20;13:15609. doi: 10.1038/s41598-023-42439-w (PMC10511714; doi:10.1038/s41598-023-42439-w)
Supplement: Supplementary file 2 — Supplementary Information 2. [file 41598_2023_42439_MOESM2_ESM.docx]

**SUPPLEMENTARY DOCUMENT**

**Using pupae as appetitive reinforcement to study visual and tactile associative learning in the Ponerine ant *Diacamma indicum***

Parth Chandak^1^ Udipta Chakraborti^1^ and Sumana Annagiri^1*^

Behaviour and Ecology Lab, Department of Biological Sciences, Indian Institute of Science Education and Research, Kolkata, Mohanpur – 741246, India

Phone: 91-33-66340000 ext 1203

*Corresponding author’s email: sumana@iiserkol.ac.in

Running title: Pupae-driven associative learning in ants

**Tactile associative learning with visually impaired ants**

**Introduction**

Vision and touch play an important role in object recognition among animals. For insects, both visual and tactile cues are essential to assess the surface they move outside or inside the nest; the touch plays a much more important role in the absence of light.

To evaluate, how prominent the association of tactile cues among ants is, in the absence of visual cues, we performed the tactile associative learning experiment with visually impaired ants.

**Methodology**

To understand tactile associative learning in ants more precisely, we removed the interference of visual cues in the ant’s performance by impairing their vision following the methodology of Mukhopadhyay and Annagiri (2021) with slight modifications; Each ant of the colony was cold anesthetized for 15 minutes using ice and the anesthetized ants were blinded with one drop of non-toxic enamel paint (Testors) using the tip of the dissection pin, carefully applied to both of their eyes. We used the same paint by which they were marked for their individual identity. After blinding all the female colony members excluding Gamergate, they were allowed to rest for 10 to 12 hours. Two hours prior to the experiment, each blinded individual was monitored to confirm that the paint on their eyes was intact and not groomed off. If any of the individuals were found to have removed the paint that particular ant was repainted to ensure that at the start of the experiment, all the members of the colony were completely visually impaired. The tactile associative learning experiment was performed by following the same methodology described in the material and methods section in the manuscript for the tactile association experiment.

**Results**

We trained 15 visually impaired ants from 5 colonies for this experiment. Out of 15 ants trained, 12 took a decision in favour of the conditioned arm during the test run [Binomial test: N = 15, k = 12, *p* =0.019]. Further, ants spent a significantly greater proportion of time on the arm with the conditioned cue (0.773±0.157) than on the arm with the non-conditioned cue (0.226±0.157) **(**Beta regression: *est.* = - 2.42, t = - 8.44, p<0.001).


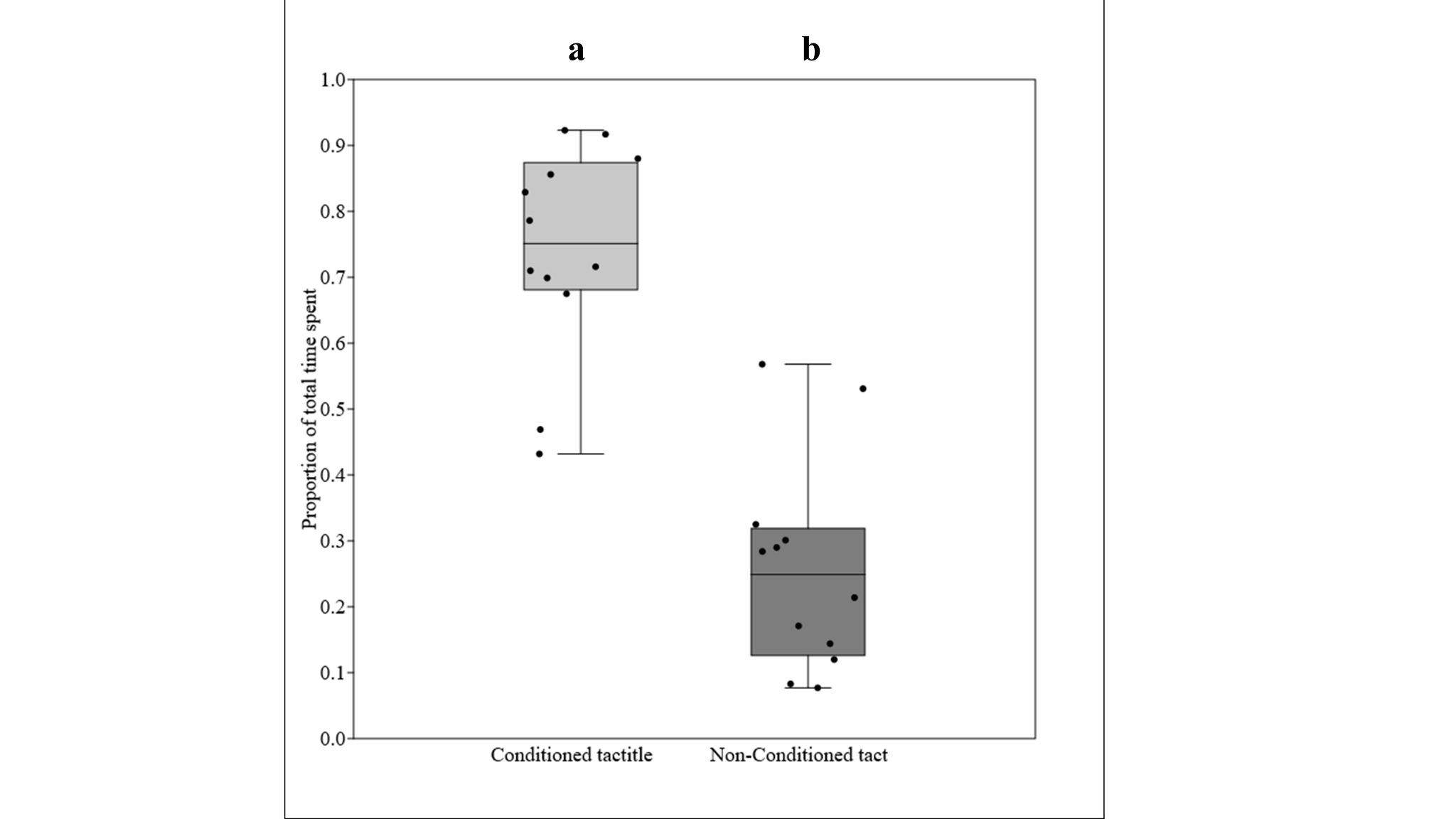


**Figure S1:** The proportion of total time spent by visually impaired ants, between conditioned and non-conditioned tactile cues has been plotted using Box-jittered box plots (N = 15). The bold black horizontal line inside the boxes represents the median, the box represents the interquartile range (IQR), and the whiskers of the boxes represent the data points that are within 1.5 x IQR. Different letters on the boxplots represent a significant difference (p < 0.05) between the two categories

**Conclusion**

In summary, visually impaired *D. indicum* ants showed associative learning for tactile cues on using pupae as the unconditioned cue. Thus, these ants were not simply visualizing the rough and smooth surface of the different arms in the current experiment (tactile association experiment) but were perceiving them as tactile cues. These findings echoes the previous experiments findings in which we found that visually impaired tandem leaders navigated to the new nest by following tactile cues from the walls of the arena. When these tactile cues were eliminated, they were unable to relocate into the new nest. However, applying paint on the ants is rather cumbersome and hence we had chosen to perform the current set of experiment with intact ants.

| **Table S1: Models for associative learning with visual and tactile cues among ants** | | | | | | | | | | | | |
| --- | --- | --- | --- | --- | --- | --- | --- | --- | --- | --- | --- | --- |
| **Experiment** | **Reward** | **Dependent**  **Variable** | **Independent**  **Variable** | **Model** | **Distribution** | **Estimate** | **Standard Error** | **t-value** | **p-value** | **Comparative tests** | **Test Value** | **p-value** |
| Tactile Associative Learning | Food | Proportion of total time spent | Non-Condition Cue | Beta Regression | Beta | -2.44 | 0.27 | -8.97 | <0.001 | Wilcoxon paired-sample test | 2.98 | 0.002 |
|  | Pupa | Proportion of total time spent | Non-Condition Cue | Beta Regression | Beta | -1.45 | 0.18 | -7.95 | <0.001 | Wilcoxon paired-sample test | 4.05 | <0.001 |
|  | Food and Pupa | Total Time spent (sec) | Pupa | Generalized Linear Model | Negative Binomial | -1.11 | 0.13 | -7.94 | <0.001 | Mann-Whitney U test | 4.71 | <0.001 |
|  | Food and Pupa | Proportion of total time spent | Pupa | Beta Regression | Beta | -0.47 | 0.23 | -2.08 | 0.037 | Mann-Whitney U test | 2.23 | 0.025 |
| Tactile Associative Learning  For visually impaired ants | Pupa | Proportion of total time spent | Pupa | Beta  Regression | Beta | -2.42 | 0.28 | -8.44 | <0.001 | Wilcoxon paired-sample test | 3.23 | <0.001 |
| Visual Associative Learning | Pupa | Proportion of total time spent | Non-Condition Cue | Linear  Model | Normal | -0.24 | 0.05 | -4.79 | <0.001 | Wilcoxon paired-sample test | 2.77 | 0.006 |
| Visual and Tactile cue conflict | Pupa | Proportion of total time spent | Conditioned with visual cue | Linear  Model | Normal | -0.32 | 0.03 | -8.62 | <0.001 | Wilcoxon paired-sample test | 4.68 | <0.001 |
| *P values considered as significant at p<0.05*  *All the independent variables with “Non-conditioned Cue” were tested against “Conditioned Cue”; “Pupa” against “Food” and “Conditioned with visual Cue” was tested against “Conditioned with tactile Cue”* | | | | | | | | | | | | |
